# Combining Two Selection Principles: Sensor Arrays Based on Both Biomimetic Recognition and Chemometrics

**DOI:** 10.3389/fchem.2018.00268

**Published:** 2018-08-02

**Authors:** Wim Cuypers, Peter A. Lieberzeit

**Affiliations:** Department of Physical Chemistry, Faculty for Chemistry, University of Vienna, Vienna, Austria

**Keywords:** electronic noses and tongues, biomimetics, molecular imprinting, aptamers, protein-based receptors, cells as sensing elements

## Abstract

Electronic noses mimic smell and taste senses by using sensor arrays to assess complex samples and to simultaneously detect multiple analytes. In most cases, the sensors forming such arrays are not highly selective. Selectivity is attained by pattern recognition/chemometric data treatment of the response pattern. However, especially when aiming at quantifying analytes rather than qualitatively detecting them, it makes sense to implement chemical recognition via receptor layers, leading to increased selectivity of individual sensors. This review focuses on existing sensor arrays developed based on biomimetic approaches to maximize chemical selectivity. Such sensor arrays for instance use molecularly imprint polymers (MIPs) in both e-noses and e-tongues, for example, to characterize headspace gas compositions or to detect protein profiles. Other array types employ entire cells, proteins, and peptides, as well as aptamers, respectively, in multisensor systems. There are two main reasons for combining chemoselectivity and chemometrics: First, this combined approach increases the analytical quality of quantitative data. Second, the approach helps in gaining a deeper understanding of the olfactory processes in nature.

## Background

### Electronic noses and tongues

The terms “electronic nose” (e-nose) and “electronic tongue” (e-tongue) are used to denote devices that detect smell and taste, respectively, similar to their mammalian counterparts. The tongue and the nose constitute chemical senses (Baldwin et al., 2011; Wilson, 2012; Cui et al., 2018; Dung et al., 2018), whereas all other perceptions, including hearing, sight, and touch, respond to physical stimuli. Mammalian sensing has several advantages which include its unique ability to distinguish odors and tastes as well as it's high sensitivity to toxic compounds such as thiols. Nonetheless, it also has some inherent limitations. Firstly, mammalian sensing is not quantitative. Secondly, both the senses are restricted to physiological conditions, thus limiting possible technological application. Thirdly, odor and taste perception varies among individuals and also depend on external factors that may lead to different results at different times for a given person. Some factors include the age and health conditions of test subjects, and environmental conditions, such as temperature, and/or smoking habits. Standardizing human olfactory and sensory data, therefore, becomes close to impossible. Finally, the olfactory sense tires over time, that is it loses sensitivity. To overcome these limitations, e-noses and e-tongues make it possible to obtain standardized, intersubjective, and quantitative information. Furthermore, they also sense analytes that are harmful to living organisms, such as toxic gases or solutions (Arshak and Harris, 2004; Baldwin et al., 2011; Wilson, 2012; Dung et al., 2018). E-noses and e-tongues usually comprise a sensor array (Shurmer and Gardner, 1992; Hong et al., 1996) which are described in the following paragraphs.

#### Electronic noses

**Figure 1:**
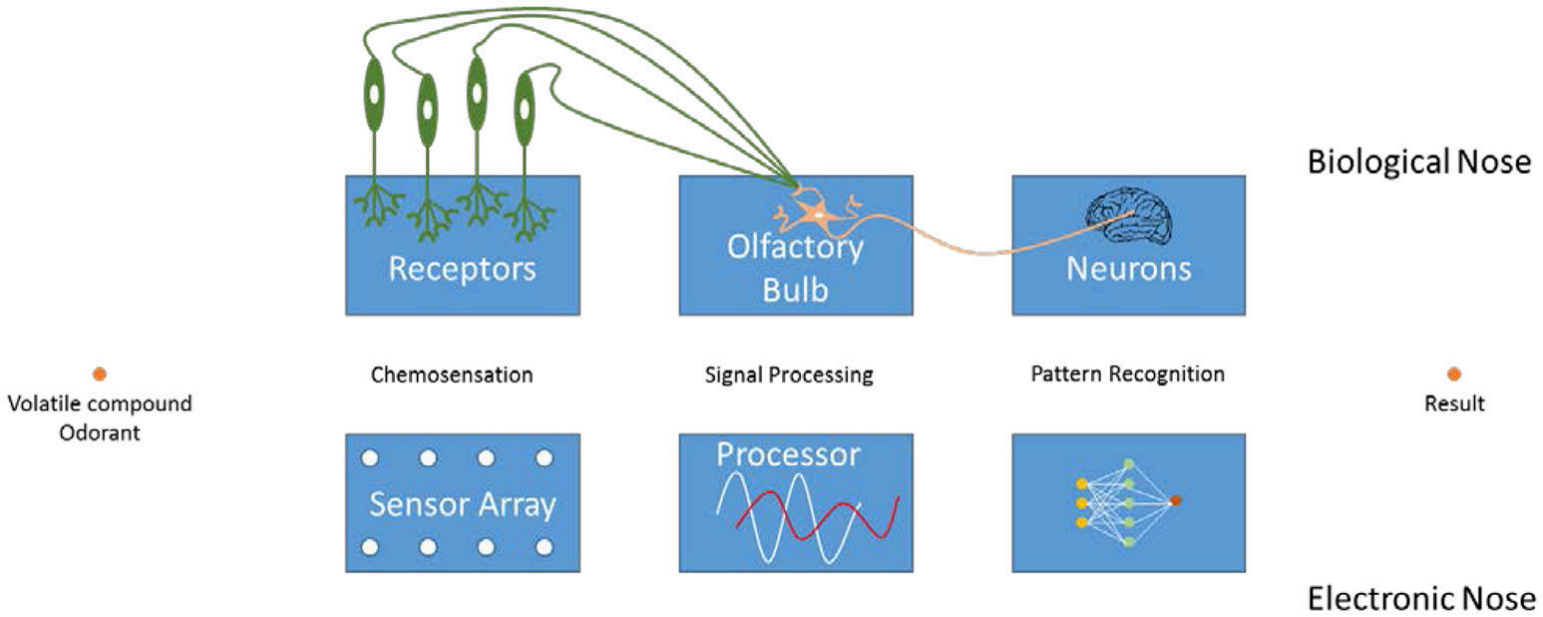
Working mechanism and comparison of electronic and biological noses.

The working mechanism of an e-nose is most conveniently explained via its natural counterpart, i.e., nose (Figure 1). First of all, the compounds from the environment are taken up by the olfactory organ of a subject. These volatile compounds (VCs) reach the olfactory epithelium, where they bind to an olfactory receptor. This generates an action potential in the respective neuron, which is transmitted to the brain. Here, the responses are collected and organized into patterns, allowing the subject to recognize the specific odorant (Schaller et al., 1998; Rinaldi, 2007; Baldwin et al., 2011; Ko and Park, 2016).

Similarly, an e-nose consists of an array of receptors that are able to bind the particular (groups of) VCs. The resulting array response is processed by using pattern recognition techniques to generate an output signal. Although individual sensors are usually not highly selective, their combined signals allow the characterization of the samples in their entirety. The main difference between natural and artificial noses is that olfaction in mammals requires a phase transition from the gaseous environment into the liquid mucus of the nose.

The use of e-noses has been reported in a wide range of applications, mostly in healthcare. Prominent areas of application are in the identification of lung cancer (Dragonieri et al., 2012; Bikov et al., 2014), kidney disorders (Di Natale et al., 1999), and heart failure (Voss et al., 2012). The food industry is another notable field of application. Controlling and monitoring ripening and spoilage processes is extremely valuable to guarantee food safety and quality (Cagnasso et al., 2010; Xu et al., 2016; Wojnowski et al., 2017). Other examples of uses of e-noses include the identification of the flavoring of wine (Macias et al., 2012) and beer (Pearce et al., 1993), fruit ripening, freshness of fish and meat (Najam ul et al., 2012), and dairy products (Gutiérrez, 2011). Environmental uses of electronic noses (Baby et al., 2000) are in water and soil quality assessments.

#### Electronic tongues

E-tongues work in liquid environments and can be compared with their human analog: The human gustatory organ contains structures called papillae, and each papilla comprises thousands of taste buds (Latha and Lakshmi, 2012). These taste buds in turn consist of 50–100 individual taste receptors. Each tastant senses differently in such a way that they have distinct mechanisms for triggering action potentials. These signals are sent to the gustatory cortex via cranial nerves which lead to pattern recognition in the brain. For an e-tongue, any substance needs to be dissolved in the liquid phase to enable detection. Binding of analytes to the distinct sensors is paired with some kind of selectivity. A unique fingerprint arises and is analyzed through pattern recognition and/or through multivariate data analysis.

### Biomimetic recognition

Biomimicry overcomes the limitations imposed by natural recognition by imitating nature and implementing its working mechanisms (Hussain et al., 2013; Hwang et al., 2015) in artificial systems. During the past few decades, substantial attention has been paid to the durability and sustainability of such “smart materials” in areas such as architecture, engineering, and medicine. For instance, one of the earliest inventions goes back to the fifteenth century when Leonardo da Vinci proposed a model of a “flying machine” based on a bird. Modern-day materials chemistry, for example, has been creating synthetic nanoscale materials such as carbon nanotubes, reverse micelles, and surfactant vesicles. Several other strategies aim at selective recognition, such as aptamers. Aptamers are (short) artificial oligonucleotides that typically comprise 20–40 nucleobases which selectively bind to defined epitopes (Gotrik et al., 2016; Zhuo et al., 2017). Finally, molecular imprinting leads to a class of biomimetic materials, known as molecularly imprinted polymers (MIPs), which attract substantial interest especially in analytics (Hussain et al., 2013). By mimicking enzyme–substrate complexes, MIPs benefit from the high selectivity between a target and a polymer.

**Figure 2:**
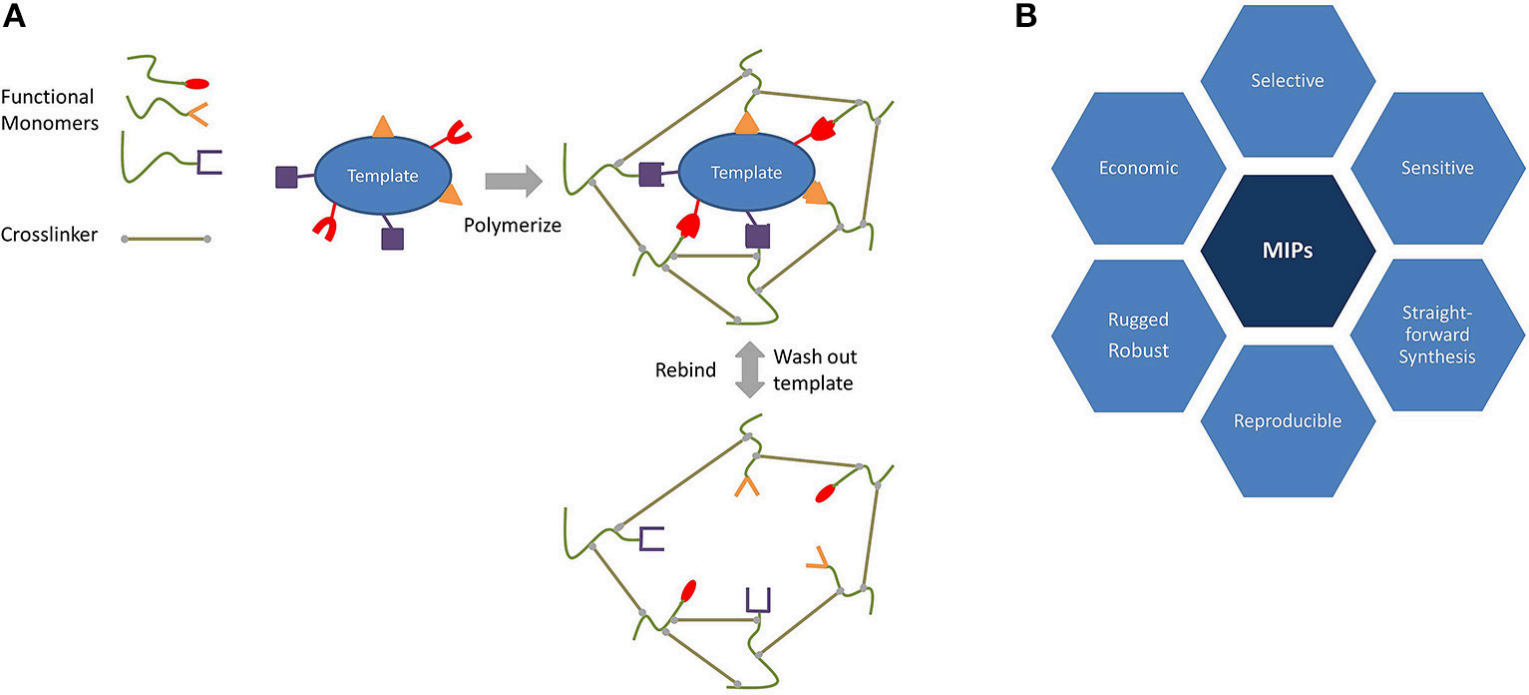
Schematic overview of **(A)** the molecular imprinting process and **(B)** its advantages.

Figure 2A sketches the imprinting process. During the first step of MIP synthesis, a target species—the template—and functional monomer(s) preform a complex (Haupt and Mosbach, 2000; Chunta et al., 2015), either via covalent bonds or noncovalent ones. The advantage of the former approach is that the template is strongly bound to a monomer, and this leads to highly selective materials with narrow affinity distribution. Removing the template from the final polymer, however, can be challenging because it involves the breaking of the said covalent bond. Noncovalent imprinting makes use of weaker (noncovalent) interactions between the functional monomer and the template, such as hydrogen bonds, dipole–dipole interactions, and van der Waals forces. These usually make it easier to remove the template from the polymeric matrix. In both the cases, the removal of the template generates cavities which are suitable for selectively recognizing the target of interest.

In addition to the functional monomer, MIPs also require crosslinking to achieve rigid, three-dimensional networks and thus to ensure stability. After polymerization, the template is finally removed from the matrix and leaves behind adapted cavities that are suitable for rebinding it. In a nutshell, it can be said that MIPs mimic natural recognition entities through the effects of binding and rebinding the targets of interest. The origin of MIP dates back to 1931, when Polyakov and his team generated silica materials which exhibited selective binding toward the solvent used during synthesis (Polyakov, 1931; Alexander et al., 2006). However, it took until the seventies and eighties of the twentieth century to establish some of the most remarkable breakthroughs by the groups of G. Wulff and K. Mosbach, respectively. Their use of organic polymers opened a whole new world for template recognition (Alexander et al., 2006).

Compared to natural species, such as enzymes or antibodies, MIPs have some fundamental advantages, as summarized in Figure 2B. These are their low production cost, long storage time, and their ruggedness. Furthermore, they are usually stable in larger ranges of pH and temperature compared to their biological counterparts. Finally, they are usually chemically more resistant and inert toward many solvents thus allowing detection of a broad spectrum of analytes, from small molecules (neurotransmitters, amino acids, and ions) to large species (bacteria, viruses, and cells). In addition, MIPs can also be synthesized for templates that cannot be addressed by antibodies or enzymes (Haupt and Mosbach, 2000; Chunta et al., 2015; Wackerlig and Lieberzeit, 2015; Chen et al., 2016).

## Implementation of MIPs in electronic noses and tongues

Electronic noses and tongues conventionally rely on low-affinity sensors and use pattern recognition to obtain selectivity, afterwards. They usually consist of arrays of said sensors, each of which is affine toward a range of analytes. It may seem counterintuitive to use highly selective receptor elements, such as MIPs, in this context. Hence, it took until the beginning of the twenty-first century to see the first publications in that area (Dickert et al., 2004; Lieberzeit et al., 2008; Iqbal et al., 2010). Those experiments were guided by the interest to achieve quantitative multianalyte sensing, rather than generating data patterns to correctly assign sensor array responses to predefined clusters.

### MIP-based electronic noses

**Figure 3:**
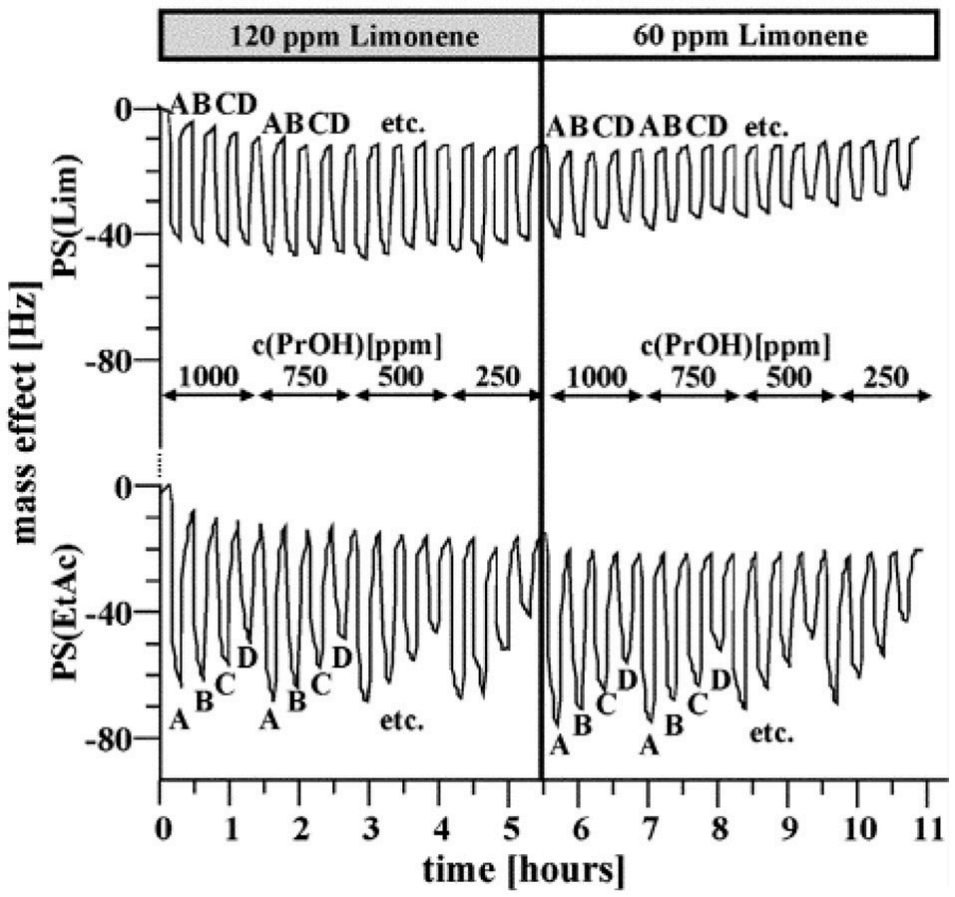
Detail of the frequency responses for the limonene sensor and the ethyl acetate sensor based on polystyrene toward different gas mixtures with the following analyte contents: limonene 120 and 60 ppm; propanol 250–1000 ppm; ethyl acetate: A: 3000 ppm, B: 2250 ppm, C: 1500 ppm, D: 750 ppm. Reproduced with permission from (Dickert et al., 2004) ^©^ RSC, Royal Society of Chemistry.

The first MIP-based electronic nose was reported in 2004 (Dickert et al., 2004). It was comprised of a device for continuous surveillance of composting processes based on a six-electrode quartz crystal microbalance (QCM) coated with both molecularly imprinted polymers and affinity materials. Polymers were chosen for their interaction properties with the respective analyte: polyurethane-based MIP toward short-chain alcohols, nonpolar polystyrene MIP toward limonene, a terpene, and two different MIPs based on each of those systems to detect acetic ester vapors to reflect their “ambivalent” functionality. Water vapor was detected by a copolymer of polyvinyl alcohol and acrylic acid leading to affinity interactions. The beauty and power of the MIP approach can be seen by the following example in detail. Although polystyrene MIP for limonene and ethyl acetate (EtAc) are chemically similar, the corresponding sensors respond differently toward those VCs, as seen in Figure 3. The EtAc sensor gives rise to a distinct response pattern determined by its vapor content, whereas this is almost invisible for the limonene MIP. Overall, the MIPs lead to a very high selectivity of individual sensors. Consequently the sensor array very accurately reproduced the time-dependent VOC patterns in the composter headspace as determined by gas chromatography–mass spectrometry (GC-MS) over long-term composting procedures, lasting for 1 week.

**Figure 4:**
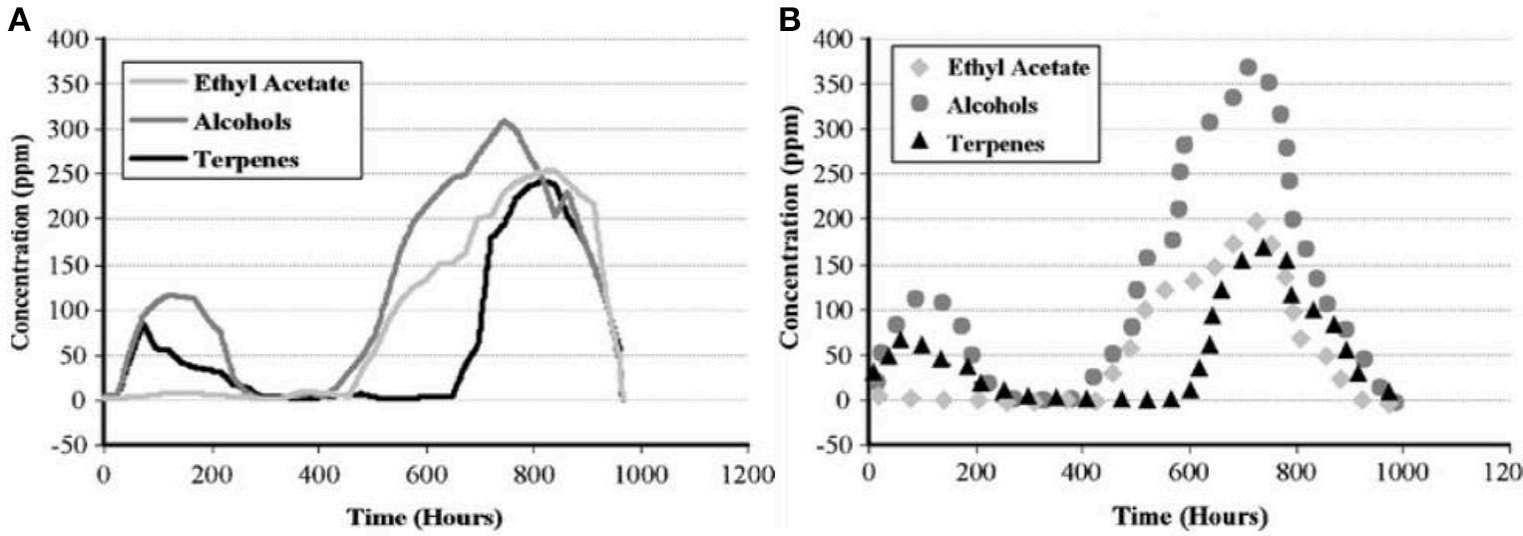
**(A)** Mass-sensitive measurements of pine decomposition. **(B)** Corresponding GC-MS validation data. Adapted with permission from Lieberzeit et al. (2008) ^©^ Springer Nature.

The real-life feasibility of the system was demonstrated in 2008 (Lieberzeit et al., 2008) through monitoring composting processes over a longer time, up to 6 weeks, after the quantitative calibration of the e-nose, which took a week. Therefore, it turned out to be possible to not only follow trend lines but also to quantify VCs, pine composting is one such example. Figure 4 shows the respective concentration profiles obtained by the e-nose and GC-MS during a measuring cycle lasting for 6 weeks.

**Figure 5:**
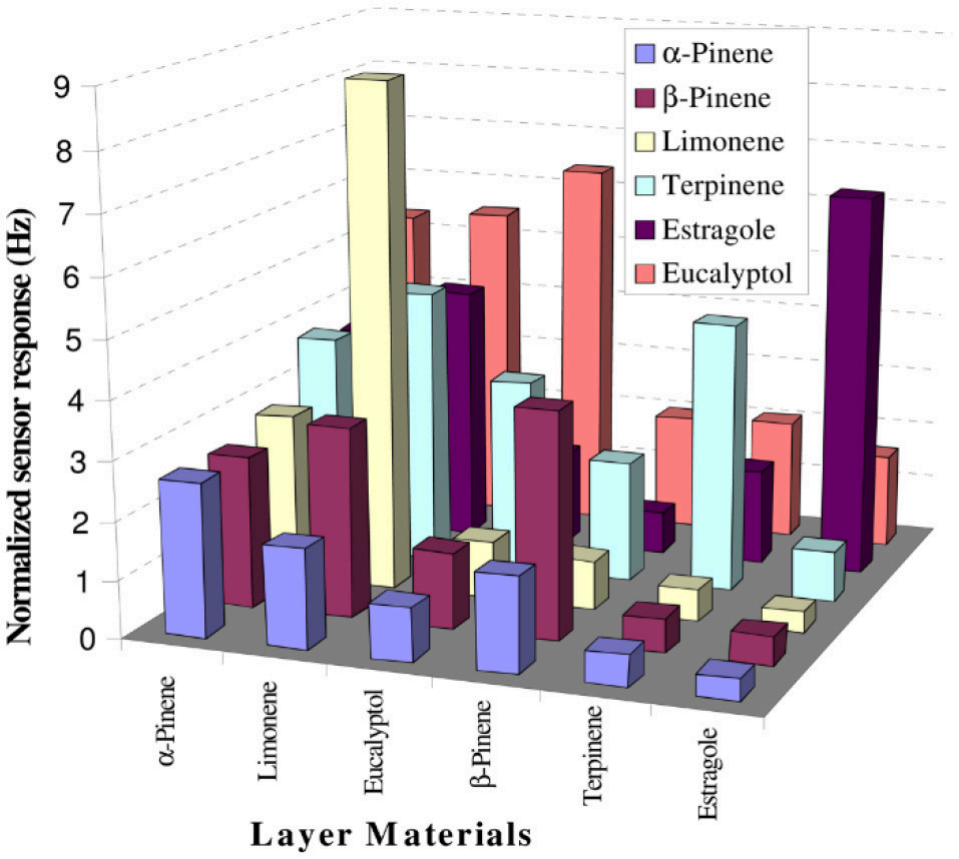
Responses of different MIP-based sensors toward terpenes. Reproduced from Iqbal et al. (2010) Creative Commons License CC-BY3.0.

Figure 5 displays the selectivity pattern of a similar set-up used for the detection and quantification of terpenes elaborated by distinct *Lamiaceae* species (Iqbal et al., 2010), namely rosemary, basil, and sage. The array allowed for distinguishing between the profiles of fresh and dried herbs, respectively.

**Figure 6:**
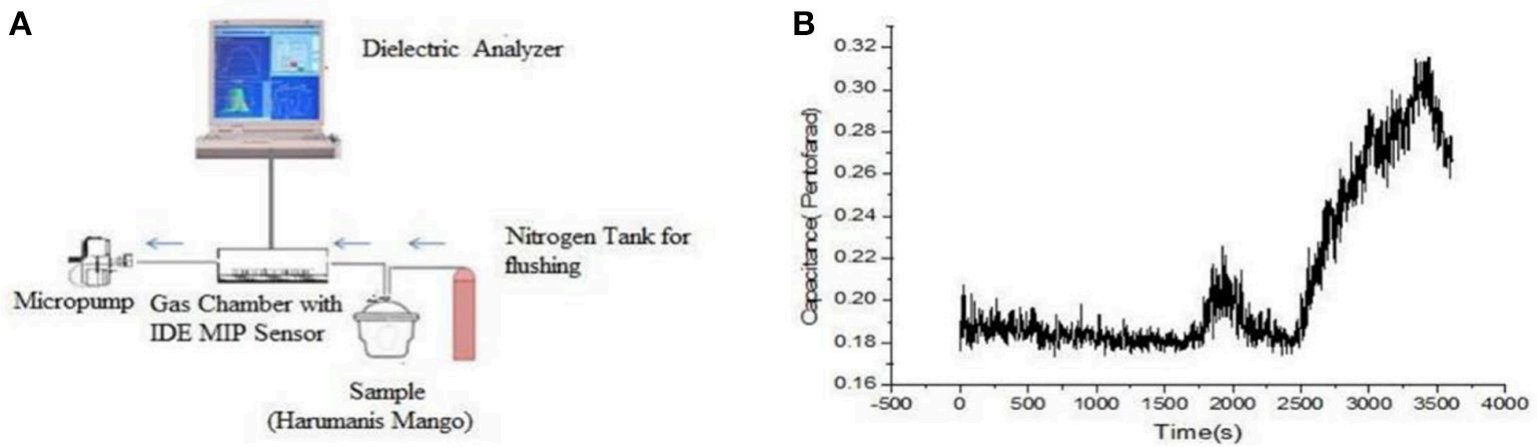
**(A)** Set-up of mango VC detection device. **(B)** Emission profile of α-pinene in time. Reproduced from Hawari et al. (2013) Creative Commons License CC-BY-NC-ND 3.0.

Hawari et al. developed a MIP-based e-nose to distinguish the ripening stages of mango during harvesting (Hawari et al., 2012, 2013) based on the detection of α-pinene emissions. Through this indicator, they defined the ripeness stage for optimal harvest maturity. Coating MIPs onto interdigital electrodes is followed by capacitance measurements. Thirty minutes after exposure to ripe mangos, the authors observed an increasing emission of pinene. However, the signal fell back a few minutes later. After 45 minutes from the start of the measurement, the signal coming from terpenes increased sharply. As seen in Figure 6, the signal decreases again 1 h after initial exposure. Because of these findings, Hawari et al. stated that α-pinene sensors had been created in a unified way such that this technique can be applied to other types of terpenes as well.

In 2016, Shinohara et al. fabricated molecularly imprinted filtering absorbents (MIFA) for sensing gas odor molecules (Shinohara et al., 2016). These MIFAs combine a high absorptive capacity with a selective filtering procedure. Because of this synergy, superior control of absorption and odor detection can be achieved. Polydimethylsiloxane (PDMS), divinyl benzene (DVB), polyvinyl alcohol (PVA), polyethylene glycol (PEG), and polyvinyl chloride (PVC) were used as absorbent materials. Absorption of ten gases and their dependence on dioctyl phthalate (DOP; a plasticizer) was measured using solid-phase microextraction (SPME) followed by GC-MS. Moreover, absorption capacities of MIFAs based on methacrylic acid (MAA) and polyacrylic acid (PAA) were evaluated toward alcohols (heptanol and nonanol) and fatty acids (heptanoic acid and nonanoic acid). Results demonstrated the superior performance of rigid MAA compared to flexible PAA in all the cases. Coupling multiple affinity sensors gives rise to discrimination of gases using pattern recognition.

Instead of using organic polymers, Liu et al. employed sol–gel materials for detecting volatile aldehyde vapors (Liu et al., 2017). These metabolic byproducts play an important role in oxidative stress as well as in cancer. Such molecularly imprinted sol–gel (MISG) materials were targeting hexanal, nonanal, and benzaldehyde and could be implemented in an e-nose system. Using a five-channel array, the three distinct vapors could be separated at low concentrations by means of principal component analysis (PCA). Finally, a randomly selected array was used for qualitative comparison.

### MIP-based electronic tongues

**Figure 7:**
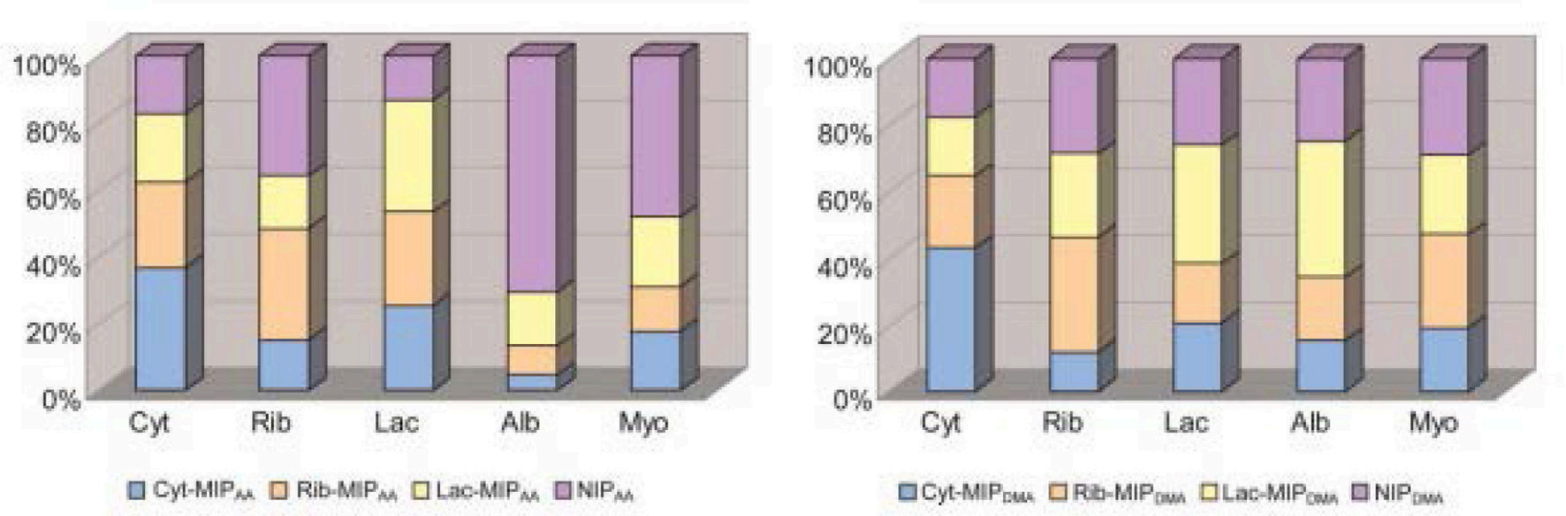
Fingerprints of five proteins tested based upon AA-based and DMA-based polymers. Cyt, cytochrome C; Rib, ribonuclease A; Lac, a-lactalbumin; Alb, albumin; Myo, myoglobin. The total amount of proteins bound corresponds to 100%. Reproduced with permission from Takeuchi et al. (2007) ^©^ RSC, Royal Society of Chemistry.

**Figure 8:**
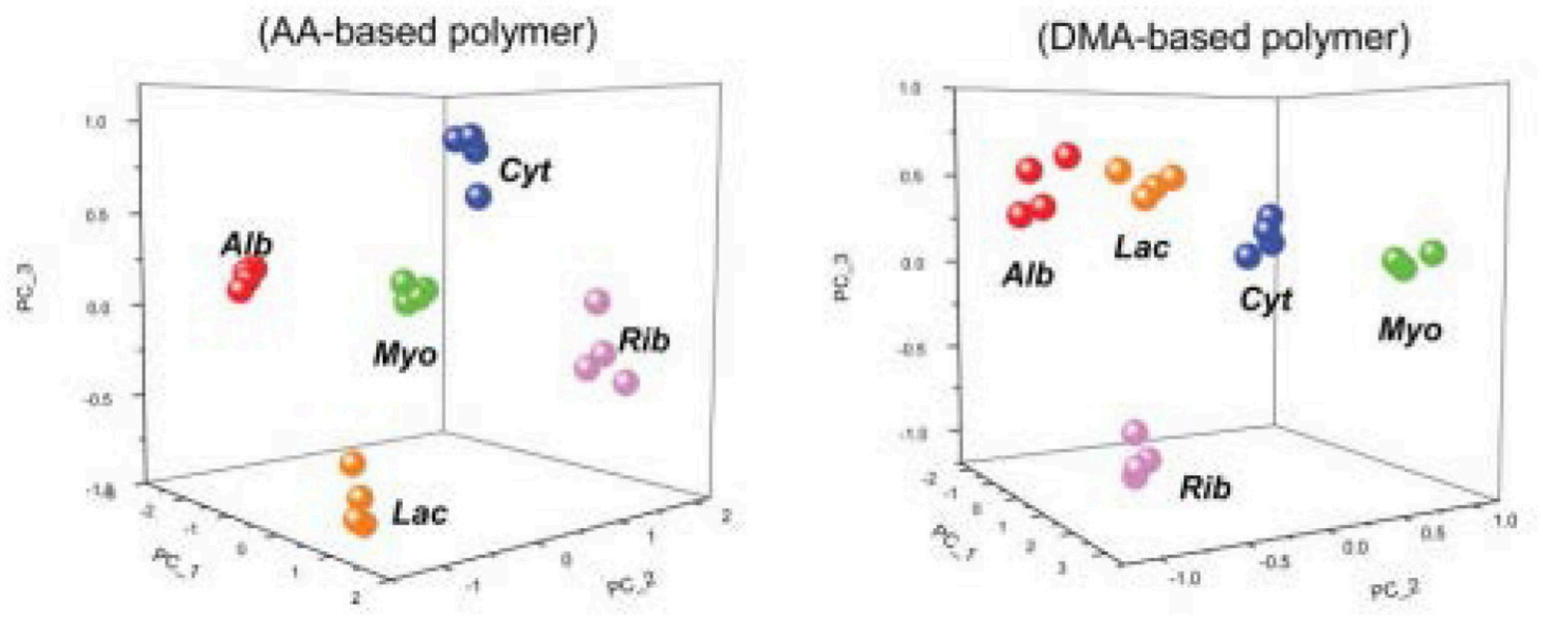
PCA score plots showing the discrimination of four trials of five different proteins based upon the bound amounts of AA-based and DMA-based polymers. Cyt, cytochrome C; Rib, ribonuclease A; Lac, a-lactalbumin; Alb, albumin; Myo, myoglobin. Alb and Myo are non-templated proteins. Alb and Myo are non-templated proteins. Reproduced with permission from Takeuchi et al. (2007) ^©^ RSC, Royal Society of Chemistry.

Using MIPs is not limited to the gas phase. Takeuchi and coworkers developed a MIP-based array to classify proteins (Takeuchi et al., 2007; Huynh and Kutner, 2015) via e-tongue in aqueous solution. They used acrylic acid and 2-dimethylaminoethyl methacrylate as functional monomers and glycosyloxyethyl methacrylate as a crosslinker. Cytochrome c, ribonuclease A, and α-lactalbumin all demonstrated the highest affinity toward their respective synthesized MIP (Figure 7). The affinities of both albumin and myoglobin were analyzed for reference. They both showed more binding toward the nonimprinted polymer than to any of the three MIPs on both the tested polymers. Figure 8 displays the PCA profiling results of these five protein groups. It turned out that the acrylate-based polymer is more suitable for correct classification of all the five proteins. It is noteworthy that albumin and myoglobin can be distinguished from the other proteins and from each other despite the fact that no MIPs were generated for either of them. This approach very clearly shows that combining “chemical” and “chemometric” selectivity indeed adds value to the system.

Feng and colleagues implemented an e-nose to distinguish clenbuterol from its metabolites: 4-aminohippuric acid (AHA) and 4-hydroxymandelic acid (HMA) (Feng et al., 2017). In 2010, clenbuterol received considerable attention in the media for its use as a doping agent in professional sports. It is a performance-enhancing drug that leads to enhanced muscle strength (George et al., 2006). In addition, it acts as a bronchodilator. Detection of very low concentrations, thus, provide a valuable tool in the battle against doping. The results showed that each MIP displays the highest affinity toward the respective target species. In this case, the authors used ethylene glycol dimethacrylate (EGDMA) as a monomer. After the imprinting process, QCM experiments were performed to determine the amount of uptake of the β-2 agonist and its analogs. This led to three distinct clusters that did not overlap; thus, distinguishing the three compounds. Overall, the method resulted in a limit of detection around LoD = 3.0 ng/mL.

## E-noses and E-tongues relying on nonpolymeric biomimetics

As previously mentioned, there is only a limited number of sensor array papers describing the use of highly selective sensors. Apart from molecularly imprinted polymers, only a few other strategies have been reported, partly relying on natural species and partly on truly biomimetic ones.

### Cells

Using living cells to generate selectivity in sensing is, comparably, a new concept. It is intriguing because it makes use of the species that is usually first exposed to a given environment. The reaction to external stimuli is rapidly followed by a response. Moreover, during this process, cells preserve their essential working mechanism. One example by Wang et al. integrates olfactory and gustatory cells with a light-addressable potentiometric sensor (LAPS) to mimic human smell and taste (Wang et al., 2007). For this purpose, olfactory neurons are grown onto the silicon dioxide chip constituting the device. The LAPS is built up in two parts, namely (1) an electrolyte insulator (SiO_2_) and (2) a semiconductor (Si). Laser light shines onto the immobilized cells. Exposure to drugs triggers action potentials that are observed through changes in the bias voltage. These are picked up by the LAPS and are converted into a corresponding photocurrent. Different concentrations (1, 25, and 50 μM) of acetic acid were added to the chip as a feasibility test to study the stimulation of the mitral cells. This resulted in concentration-dependent elevated frequency responses upon addition of acetic acid solutions. Next, taste buds were successfully placed onto LAPS chips. Four basic tastants, namely sodium chloride (NaCl) for “salty,” hydrochloric acid (HCl) for “sour,” sucrose for “sweet,” and magnesium sulfate (MgSO_4_) for “bitter,” were administered. Changes in photocurrent underwent fast Fourier transformation (FFT) analysis. Characteristic peaks resulting from this approach contain information about the corresponding taste profile. Very recently, a report showed a very special case of cell-based e-noses by linking a microelectrode array to the olfactory bulb of a mouse *in vivo* (Gao et al., 2018). In a similar way, the authors achieved high selectivity and sensitivity toward odorants containing benzene rings that reached down to 10^−5^M in the case of trinitrotoluene (TNT). However, the extent to which such an approach can still be regarded “biomimetic” is of course questionable.

### Enzymes

It is well known that enzymes can be implemented as recognition elements in sensors. The blood-glucose sensor based on glucose oxidase is the most famous example of such an approach. Although many research groups have reported enzymatic biosensors so far, only few of them are implemented into e-noses or e-tongues.

As a first example, Keller and his group developed an amperometric enzyme-based biosensor to detect umami by monitoring the amount of L-glutamate in tomato specimens (Pauliukaite et al., 2006). L-glutamate oxidase was linked onto an electrode followed by exposing the sensor to increasing concentrations of glutamate. This led to concentration-dependent sensor signals and a detection limit of 50 μM. Combining multiples of these biosensors would overcome the restriction to quantitative detection. It opens up the opportunity for qualitative measurements, i.e., discrimination among different tastants.

An already established enzyme-based e-tongue was developed by Busch et al. (2006). Tyrosinase and peroxidase were used to verify the presence or absence of phenolic compounds that are characteristic for bitterness in virgin olive oil. Correlation coefficients of 0.82 and 0.87 could be achieved for describing the relationship between tyrosinase- and peroxidase-based biosensors on the one hand and phenol content on the other. Moreover, measurements were highly repeatable, revealing a residual standard deviation (rsd) of 6%.

In 2005, Dock and his group developed an e-tongue to assess wastewater quality through chemometrics (Tønning et al., 2005) based on enzymatically modified screen-printed platinum electrodes. The sensor system targets different factors, such as chemical oxygen demand (COD) and inhibition of nitrification. Furthermore, enzyme-based amperometry forms the basis of detection. Combining an eight-sensor array together with PCA leads to clear discrimination between four different wastewater samples (untreated, alarm, alert, and normal water). The temporal drift of individual sensor signals could be overcome by using the entire array's response pattern.

### Proteins and peptides

Most recognition elements in nature are based on proteins; hence, using them in sensors is also logical. The best examples are, of course, enzyme electrodes and immunosensors. However, there is a beautiful example for mimicking human olfaction. In 2012, Lee et al. published the development of a human-like nanobioelectronic nose with comparable sensitivity and selectivity as its natural counterpart (Lee et al., 2012). They incorporated olfactory receptor proteins onto carboxylated polypyrrole nanotubes (CPNTs) and performed resistance measurements on these systems. They achieved a detection limit of approximately 0.02 parts per trillion (ppt) for helional gas. Hence, detecting gaseous molecules in a similar fashion as the human nose was feasible.

It is well-known that the functionality of most proteins is located on a clearly defined fraction of the respective chain/structure. Hence, constructing sensors based on short peptide chains is possible. An example of an e-tongue based on such peptide structures has been reported for detecting different dioxins (Mascini et al., 2004); in this case, biomimetic traps comprising pentapeptides were developed and coated onto QCMs. The corresponding sensor array determined the components of a mixture comprising three dioxins in a highly selective manner in a concentration range of 1–20 parts per billion (ppb). The main focus of the system in this case was to determine several dioxins simultaneously, rather than undertaking sequential measurements.

### Aptamers

**Figure 9:**
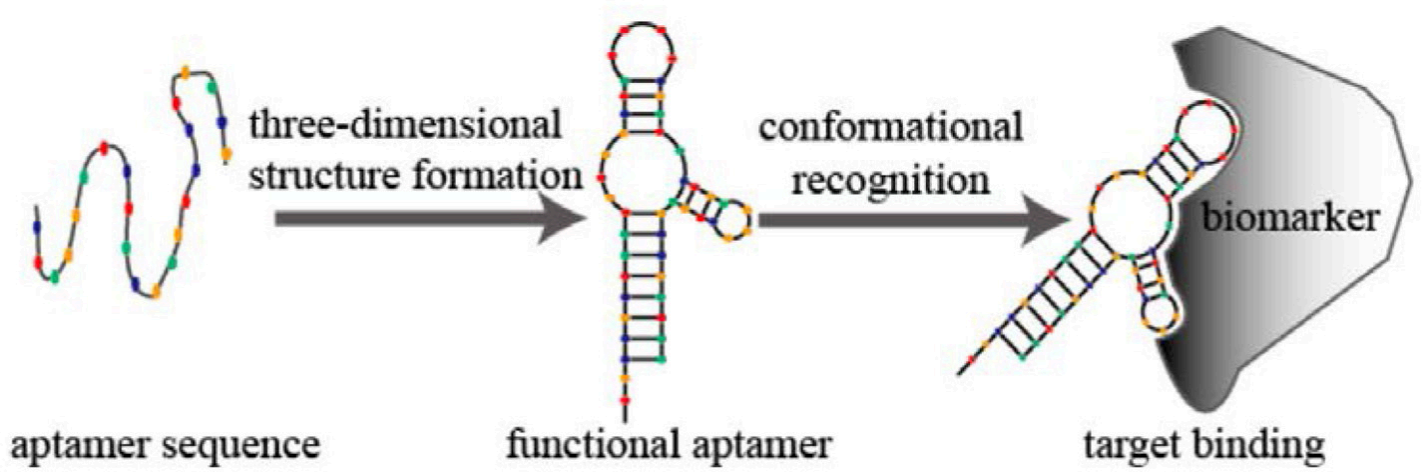
Schematic diagram of aptamer conformational recognition of targets to form an aptamer–target complex. From Sun and Zu (2015), CC-BY.

The overwhelming majority of aptamers comprises RNA oligomers or single-stranded DNA oligomers that are less than a hundred nucleobases long (Eifler, 2014; Zhuo et al., 2017). Aptamers are produced through the “selection of ligands by exponential enrichment,” abbreviated as SELEX (Sun and Zu, 2015; Zhuo et al., 2017). Figure 9 describes the working mechanism of aptamers; they bind their targets with comparable selectivity similar to antibodies binding the antigens. Within the scope of this review, Eifler (2014) reported an electronic nose in combination with a biosensor for the detection of deoxynivalenol (DON). DON is a toxic secondary metabolite released by *Fusarium* species. In a first step, an electronic nose, based on metalloporphyrins, was established to detect DON. Cross-validation studies showed that the correct classification rate between infested and noninfested species reached 83%. In addition, it was feasible to distinguish between two levels of infections and between two fungal species with classification rates of 91% and 94%, respectively. However, this approach was not strong enough to quantitatively determine the DON levels. Therefore, a 78-base aptamer was selected and implemented to bind DON, despite other molecular species interfering. This made it possible to quantitatively detect DON, despite very strong matrix effects. In conclusion, one can say that, in the concrete case, only the combined effort of a sensor array and a selective sensor is allowed for achieving the necessary selectivity and sensitivity.

In a similar approach, an array consisting of ion-selective field-effect transistors (ISFETs) coated with aptamer was used to detect vanillin in foods and beverages without sample pretreatment (Andrianova et al., 2017). Vanillin is not only useful in fragrance and flavoring but can also evoke allergic reactions, making the need for detection inevitable (van Assendelft, 1987). At first, hybridized DNA was present on an ISFET surface. Addition of vanillin led to the dissociation of the DNA probe. The outcome was a concentration-dependent change in the surface potential. Through this method of signal amplification, a 15-fold better limit of detection could be reached. As already discussed in the previous case, the strength of the system lies in the fact that multiple sensors are used to generate a signal.

Du et al. (2013) reported odor detection using a piezoelectric biosensor coated with an olfactory receptor-based tag aptamer. First, a QCM surface was coated with anti-His towards the His-tagged olfactory receptor of *C. elegans*, ODR-10. QCM results revealed high binding responses of the sensor toward diacetyl, the natural ligand of ODR-10. This example can be the starting point of artificial designed arrays that can detect desirable volatiles, opening up very interesting potential applications in mimicking olfaction.

## Conclusions and outlook

Overall, only a very limited number of sensor arrays published use selective detection. A short Scopus research report, as of June 2018, revealed more than 100,000 publications dealing with “chemical sensor,” almost 6,000 for “electronic nose,” and only some 270 for “electronic nose” combined with “highly selective.” Of these, less than 200 papers reported on combining molecular imprinting or biomimicry with electronic noses or tongues. Expanding the search toward the entire supramolecular analytical chemistry reveals that the topic of this review covers only a small yet an important part of sensor science. This seems logical because the rationale behind developing electronic noses and tongues is to use sensors with broadband chemical response and generate selectivity *in silico* via chemometric treatment afterwards. When aiming at sample classification, for instance, when recognizing/distinguishing odors, such an approach indeed makes sense. The main application scenario for arrays comprising inherently highly selective sensors is to detect multiple analytes in a simultaneous manner. Indeed, most examples for such e-noses in the literature have exactly that goal in mind, whether it may be by detecting different dioxins or for aiming at different tastants. In terms of future potential, aiming at implementing cells/tissues into sensor arrays similarly to the existing e-noses and e-tongues is highly interesting. The detailed recognition mechanism remains unclear in this case. Useful information, thus, requires chemometric analysis of the data. However, the approach allows using the responses of exactly the type of cells that first come into contact with a given pollutant in the living systems. Therefore, such cells are most useful to mimic first response *in vivo* without the need for carrying out animal experiments. This does not only lead to more reliable data but also circumvents ethical issues related to animal experiments. A slightly different and ethically more problematic approach aims at interfacing the actual (mammalian) olfaction organs with an artificial sensor array to use the respective animal as recognition “species.” Although it is interesting from the scientific point of view due to the deeper insight into the processes of olfaction and tasting, applying such approaches on a commercial scale is of course impossible. Finally, one could think about an application scenario, in which natural recognition is fully replaced by biomimetic one. This would open up ways to establish bioassays—e.g., for assessing toxicological parameters—on fully artificial systems. The beauty of the approach—especially compared to single-sensor measurements—lies in the fact that this would allow for testing the influence of a certain species toward a range of targets and (bio) receptors.

## Author contributions

WC undertook the initial literature research and wrote the first version of the manuscript after having discussed the outline/structure with PL. He also participated in interative steps on earlier version of the manuscript. PL laid out the general structure of the article, corrected, and extended versions, as well as added literature and topics and prepared the final edited version of the manuscript.

## Conflict of interest statement

The authors declare that the research was conducted in the absence of any commercial or financial relationships that could be construed as a potential conflict of interest.
